# Precision modeling of mitochondrial diseases in zebrafish via DdCBE-mediated mtDNA base editing

**DOI:** 10.1038/s41421-021-00307-9

**Published:** 2021-09-03

**Authors:** Jiayin Guo, Xue Zhang, Xiaoxu Chen, Haifeng Sun, Yichen Dai, Jianying Wang, Xuezhen Qian, Lei Tan, Xin Lou, Bin Shen

**Affiliations:** 1 State Key Laboratory of Reproductive Medicine, Nanjing Medical University, Nanjing, Jiangsu China; 2 Gusu School, Nanjing Medical University, Nanjing, Jiangsu China; 3 Model Animal Research Centre, Medical School of Nanjing University, Nanjing, Jiangsu China; 4 Center for Global Health, School of Public Health, Nanjing Medical University, Nanjing, Jiangsu China; 5 Women’s Hospital of Nanjing Medical University, Nanjing Maternity and Child Health Care Hospital, Nanjing Medical University, Nanjing, Jiangsu China

**Keywords:** Genomic analysis, Biological techniques

Dear Editor,

Mitochondrial diseases are a group of heterogeneous genetic disorders that are characterized by dysfunctional mitochondria and often manifest as severe even lethal condition^1^. Mitochondrial diseases can arise from mutations affecting either the nuclear gene or the mitochondrial DNA (mtDNA), whereas pathogenic mutations in mtDNA more commonly cause mitochondrial disease than mutations in nuclear genes do^2^. Up to now, more than 270 pathogenic variants of mtDNA have been reported^3^. Animal models which precisely install these variants are urgently needed both to deepen our understanding for the etiology of mitochondrial diseases and to develop therapeutic strategies. The development of such models, however, has been hindered by the technical challenge of editing mtDNA in a programmable fashion. In recent years, some progresses have been made for mitochondrial genome engineering. Re-engineered RNA-free programmable nucleases such as mtZFN and mitoTALEN function effectively in mitochondria^4,5^. In a recent work, researchers harnessed a bacterial cytidine deaminase, DddA, to archive precise manipulation of mtDNA sequences^6^. In this system, the domain containing 1264–1427 amino acids of DddA (DddAtox) was split into two inactive halves, which were fused with transcription activator-like effector (TALE) and a uracil glycosylase inhibitor, resulting in DddA-derived cytosine base editors (DdCBEs). Besides the study in tissue culture system, installation of disease-associated mtDNA variants in mouse with this DddA_tox_-TALE strategy has been reported lately^7^. Acclaimed for multiple research advantages and flexibility of manipulation, zebrafish is an attractive animal model for studying human disease^8^. The ability to generate pathogenic mtDNA mutations in zebrafish could accelerate the researches ranging from mitochondrial biology, drug discovery to therapeutic approaches development.

In the current study, we set out to utilize the TALE-DddA_tox_ fusion deaminases to establish a method for generating models of zebrafish mitochondrial diseases. At first, a variable di-residue (RVD) plasmid library including 192 modules, as well as 4 MTS-DdCBE (mitochondrial localization) and 4 NLS-DdCBE (nuclear localization) backbone plasmids, were constructed (Supplementary Fig. S1a–c). With this library, DdCBE expression plasmids for any desired target site can be obtained within 36 h by one-step Golden Gate assembly (Fig. 1a). To verify the activity of DdCBE, we generated 4 pairs of MTS-DdCBE for the reported ND1 site (Supplementary Fig. S2a), including Left-G1333C (L1333C) + Right-G1333N (R1333N), L1333N + R1333C, L1397C + R1397N and L1397N + R1397C. First, we examined the subcellular localization of the L1333C + R1333N pair in HEK293FT cells and found that they could successfully locate to mitochondria (Supplementary Fig. S2b). Then we transfected cells with each DdCBE pair and found that the ratio of C-to-T conversion increased over time (Supplementary Fig. S2c). To quantitatively evaluate the editing efficiency of DdCBE pairs, deep sequencing was performed. The results showed that different split types and orientations of DddA_tox_ could mediate variable editing efficiencies, with the highest efficiency up to 80.12 ± 3.76% (Supplementary Fig. S2d). These results demonstrate that DdCBE pairs generated by our RVD library can facilitate efficient mtDNA editing in human cell. To generate zebrafish models for mitochondrial diseases, we selected 5 pathogenic loci within 5′-tC motif on human mtDNA (G8363A, G3733A, G13513A, G12276A and G3376A), and designed DdCBE pairs to target the corresponding zebrafish loci (G8892, G4247, G14076, G12833 and G3890) (Supplementary Fig. S3). To screen highly efficient DdCBE editors, the pEGFP-N1 plasmids harboring the target fragments and NLS-DdCBE pairs were constructed and used to co-transfect HEK293T cells. Notably, the L1397C + R1397N combination yielded the highest activity at G8892, G4247 and G14076 sites, with 91.28 ± 1.04%, 72.27 ± 1.96%, and 85.93 ± 0.33% editing efficiencies (Supplementary Fig. S4a–c). However, DdCBE pairs targeting G12833 and G3890 showed low C ∙ G-to-T ∙ A conversion rate and were discarded. NLS-DdCBE pair showing the best performance for each site was selected to make MTS-DdCBE constructs, and the constructs were then in vitro transcribed to mRNAs by a pre-set T7 promoter. We first evaluated the toxicity of DdCBE to zebrafish embryos by injecting DdCBE pair mRNAs into 1-cell zygote. Dosage-depended non-specific toxicity was observed in all DdCBE pairs (Supplementary Table S1) and the injection dose for DdCBE mRNA was fixed at 100 pg/embryo for all the subsequent experiments. To examine the stability of DdCBE protein in fish embryo, mRNAs of G8892 DdCBE pairs were injected and protein samples from different stages were analyzed. The result indicated, by injection of mRNA, time window of DdCBE editing in fish embryo spanned from 12 to 48 hpf (Supplementary Fig. S4d). By injecting mRNAs of all four MTS-DdCBE pairs of editors for G8892 to embryos, we found that the L1397C + R1397N pair also worked best in zebrafish embryos (Supplementary Fig. S4e), corroborating that the screening strategy in HEK293FT cells is effective for zebrafish mtDNA editing.

**Fig. 1:**
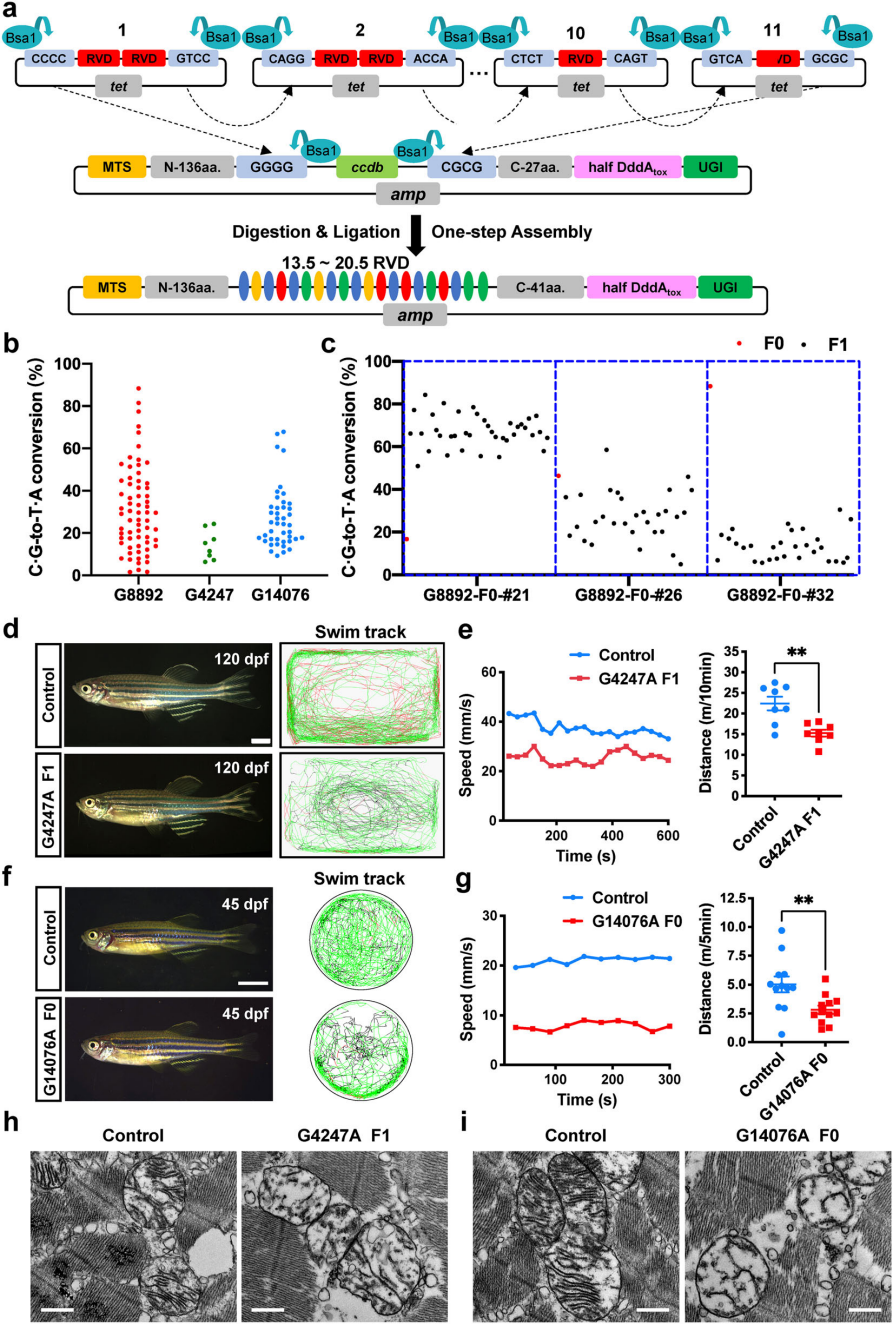
DdCBE-mediated mtDNA base editing in zebrafish. **a** Schematic overview of one-step assembly of DdCBE. Top, plasmids containing intended RVD are cleaved using *Bsa*I to produce sticky ends. Middle, the RVDs with sticky ends are allowed to ligate to backbones sequentially. Bottom, the assembled DdCBE vectors contain 13.5–20.5 RVDs. Twenty-seven amino acids (aa) are put after ccdb in the backbone, and 14 aa are put in half RVD module. There are a total of 41 aa at C-terminus of TALE after assembly. **b** Deep sequencing analysis of C ∙ G-to-T ∙ A conversions in G8892, G4247 and G14076 founders. **c** Deep sequencing analysis of transmission of mtDNA mutation in offspring of G8892 founders. Red dot and black dot indicate individual zebrafish of F0 and F1, respectively. **d** Left panels, live images of control and G4247A F1 zebrafish at 120 days post-fertilization. Lateral view, anterior to the left. Scale bar, 2 mm. Right panels, representative swim tracking of individual animals. Color coding represents movement speed: red, faster than 75 mm/s; green, 25–75 mm/s; black, slower than 25 mm/s. **e** Quantification of total distance and speed of G4247A F1 zebrafish at 120 days post-fertilization (means ± SEM, *n* = 8 for each group). Significance was calculated with unpaired two-tailed Student’s *t*-test (**P < 0.01). **f** Left panels, live images of control and G14076A founder zebrafish at 45 days post-fertilization. Lateral view, anterior to the left. Scale bar, 2 mm. Right panels, representative swim tracking of individual animals. **g** Quantification of total distance and speed of G14076A founder zebrafish at 45 days post-fertilization (means ± SEM, *n* = 12 for each group). Significance was calculated with unpaired two-tailed Student’s *t*-test (**P < 0.01). **h**, **i** TEM images of skeletal muscle from F1 G4247A and F0 G14076A fish demonstrating abnormal mitochondrial ultrastructure. Scale bar, 500 nm.

To generate zebrafish with desired mtDNA variants, mRNAs of the MTS-DdCBE pair (L1397C + R1397N) with the best performance were injected into 1-cell zygote. First, total DNA from pooled embryos (including mtDNA) was prepared at 48 hpf and analyzed, the results showed that G-to-A conversion could be successfully detected at G8892, G4247 and G14076 (Supplementary Fig. S4f). Encouraged by these results, we individually cultured injected embryos and collected the skin cells at day 4 post-fertilization for genotyping. As a result, 82.5% (66/80) G8892 samples, 26.7% (8/30) G4247 samples and 59.7% (43/72) G14076 samples were successfully edited (Supplementary Table S2). The G-to-A conversion rate in each fish was variable and in some samples was high enough to be recognized as A on sequencing chromatogram (Supplementary Fig. S4g). To further quantify the mutation load of each edited fish, we performed deep sequencing and found that the editing frequencies ranged from 5.78% to 88.32% for G8892A, from 6.36% to 23.48% for G4247A and from 9.31% to 67.9% for G14076A (Fig. 1b; Supplementary Table S2). To further characterize the editing status in founder fish, we collected major tissues and organs from two male founders. Sequencing results revealed a mosaic pattern of G-to-A conversion throughout the fish body (Supplementary Fig. S5a, b). This chimerism could be caused by rapid cleavage in early stage embryos. Then we asked whether the DdCBE-mediated mtDNA mutation is heritable. F1 offspring were collected from 6 female G8892 founders and 2 female G4247 founders by out-crossing with wild-type male fish. The sequencing results showed that 5 out of 6 founders could transmit G8892A mutation to their offspring, and the edited embryos could compose up to 72.5% (29/40) of the offspring (Supplementary Fig. S6a and Table S3). Two G4247 founders also transmitted the desired editing to their offspring successfully (Supplementary Fig. S6b and Table S3). We next quantified the mutation loads of positive F1 embryos and the deep sequencing result showed G8892A F1 harbored edited mtDNA from 1.33% to 84.33%, and G4247 F1 harbored edited mtDNA from 6.32% to 40.42% (Fig. 1c; Supplementary Fig. S6c–f and Table S3). We also noticed in some cases, mutation loads widely diverged between founders and their offspring (Fig. 1c; Supplementary Fig. S6c–f and Table S3). These results demonstrate that DdCBE-mediated mtDNA editing can be inherited and the mutation load in offspring could potentially exceed the pathogenic threshold, which encourage us to examine the mitochondria disfunction phenotype in F1 animals.

To examine whether zebrafish is suitable for modeling mitochondrial diseases, we investigated the consequences of introducing disease-associated mtDNA variants. Human genetics research indicated that E143K mutantion for *MT-ND1* gene is pathogenic in Leber’s hereditary optic neuropathy (LHON)^9^, so we first analyzed G4247A fish. F1 G4247A fish with at least 25% mutation load did not display perceivable morphology or behavior defect during development (Supplementary Fig. S7a, b). Since progressive physiological deterioration is common in human patients, we re-examined the F1 G4247A fish at 120 days post-fertilization and detected defective motility by swim track assay (Fig. 1d, e). Previous sequencing results revealed that our method could reach the editing efficiency high up to 88.32% in F0 fish (Supplementary Table S2), which prompted us to inspect the F0 animals for mitochondria disfunction phenotype. Multiple clinical cases have linked the D393N mutantion for *MT-ND5* gene to Leigh syndrome and MELAS^10^, and our method could attain up to 67.9% editing efficiency in G14076A F0 fish. We examined the F0 G14076A fish with over 35% mutation load at 45 days post-fertilization and discovered significant compromise of motility (Fig. 1f, g). Abnormal mitochondrial morphology and ultrastructure is a common phenotype observed in mitochondrial disorder patients bearing mtDNA mutation. To examine whether the edited fish could recapitulate this pathogenic feature, we collected fish skeletal muscle samples and analyzed them with transmission electron microscopy (TEM). Evident cristae degeneration and fragmentation in the mitochondrial matrix were identified in F1 G4247A and F0 G14076A samples (Fig. 1h, i). Among the mtDNA editing fish lines we successfully generated in this study, we noticed no obvious phenotype for G8892A F0 and F1 fish, even though the mutation load is higher than 80% in some animals (Supplementary Fig. S7c–f). This interesting observation indicated that more studies are needed to further characterize the pathogenicity of this variant. These results establish that precise DdCBE editing in fish can be applied to model mitochondrial diseases and with our method, F0 phenotyping is feasible.

To profile the off-target activity of DdCBE, we performed whole mtDNA re-sequencing for founders for each site. Notably, most off-target events were centered around the on-target sites in G8892 and G14076 founders (Supplementary Fig. S8a–c), suggesting that the off-target editing may be induced by unstable binding of DdCBE pair. In addition, the sparse distant off-target events along the whole mtDNA could be detected (Supplementary Fig. S8a–c), which may be caused by sequence- independent activity of DdCBE. Among the off-target editing, almost all off-target sites (OTS) were detected with G-to-A and C-to-T conversions (Supplementary Fig. S9a–d). The average frequencies of mitochondrial genome-wide off-target editing by G8892 and G4247 DdCBE were similar to the baseline signal in wild-type fish, whereas G14076 DdCBE showed higher off-target editing frequency (Supplementary Fig. S9e). To further characterize the off-target effects induced by DdCBE, OTS with conversion rate over 1% in any sample were further analyzed. Besides a strong preference for 5′-tC context, we found that C within 5′-aC, 5′-acC and 5′-cC motif could be modified by DdCBE (Supplementary, Fig. S8d, e). Taken together, DdCBE can mediate precise base editing in zebrafish mtDNA with relatively low off-target editing.

## Supplementary information

Supplementary information

## Data Availability

The high-throughput sequencing data have been deposited to the NCBI Sequence Read Archive (SRA) database (accession ID, PRJNA721862).

## References

[1] Craven L, Alston CL, Taylor RW, Turnbull DM (2017). Recent advances in mitochondrial disease. Annu Rev. Genomics Hum. Genet.

[2] Tuppen HA, Blakely EL, Turnbull DM, Taylor RW (2010). Mitochondrial DNA mutations and human disease. Biochim Biophys. Acta.

[3] Lott, M. T. et al. mtDNA variation and analysis using mitomap and mitomaster. *Curr. Protoc. Bioinform.* **44**, 1.23.1–26 (2013). 10.1002/0471250953.bi0123s44 PMC4257604 25489354

[4] Bacman SR (2018). MitoTALEN reduces mutant mtDNA load and restores tRNA(Ala) levels in a mouse model of heteroplasmic mtDNA mutation. Nat. Med.

[5] Gammage PA, Rorbach J, Vincent AI, Rebar EJ, Minczuk M (2014). Mitochondrially targeted ZFNs for selective degradation of pathogenic mitochondrial genomes bearing large-scale deletions or point mutations. EMBO Mol. Med.

[6] Mok BY (2020). A bacterial cytidine deaminase toxin enables CRISPR-free mitochondrial base editing. Nature.

[7] Lee H (2021). Mitochondrial DNA editing in mice with DddA-TALE fusion deaminases. Nat. Commun..

[8] Bradford YM (2017). Zebrafish models of human disease: gaining insight into human disease at ZFIN. ILAR J..

[9] Valentino ML (2004). The ND1 gene of complex I is a mutational hot spot for Leber’s hereditary optic neuropathy. Ann. Neurol..

[10] Shanske S (2008). The G13513A mutation in the ND5 gene of mitochondrial DNA as a common cause of MELAS or Leigh syndrome: evidence from 12 cases. Arch. Neurol..

