# Supplementary material for: Precision modeling of mitochondrial diseases in zebrafish via DdCBE-mediated mtDNA base editing
Source: Cell Discov. 2021 Sep 3;7:78. doi: 10.1038/s41421-021-00307-9 (PMC8417030; doi:10.1038/s41421-021-00307-9)
Supplement: Supplementary file 1 — Supplementary information [file 41421_2021_307_MOESM1_ESM.pdf]

Supplementary information

Precision modeling of mitochondrial diseases in zebrafish via DdCBE-mediated mtDNA base editing

Contents

Materials and Methods

Figure S1. Architecture of RVD library and DdCBE backbone.

Figure S2. Activity of one-step assembled DdCBE pair in HEK293FT cells.

Figure S3. Conservation analysis of targeting sites between human and zebrafish.

Figure S4. DdCBE-mediated mtDNA mutations in zebrafish.

Figure S5. DdCBE-mediated editing in tissues from founders.

Figure S6. Germline transmission of mtDNA base editing.

Figure S7. Morphology feature and behavior analysis of mtDNA edited fish.

Figure S8. Off-target analysis by whole mtDNA sequencing.

Figure S9. Off-target analysis in founders.

Table S1. Toxicity of DdCBE in zebrafish embryogenesis.

Table S2. Summary of mtDNA base editing efficiency of founders.

Table S3. Summary of germline transmission in offspring.

Table S4. Primer information.

Supplementary sequence

Materials and Methods

Construction of RVD library

We designed 10 staggered positions for Dimer RVD modules (Position 1-10), 4 staggered positions for monomer RVD modules (Position 7-10) and half modules (Position 8-11), respectively. Each module flanked with two Bsa I sites was amplified from the reported library [1] and cloned to the vector containing tetracycline resistance gene [2], creating a set of 192 module plasmids. Backbone plasmids MTS-DdCBE and NLS-DdCBE, with localization signal, N terminus of TALE, ccdb, C terminus of TALE and split DddA, were synthesized according to the previous reports [1, 3]. The complete set of plasmids for assembling DdCBE has been deposited to Addgene (Shen Lab DdCBE kit).

Assembly of DdCBE

To construct DdCBE expression plasmids, 50 ng of each module and 100 ng of backbone plasmid were subjected to digestion and ligation in a single 10 µL reaction containing 0.5 µL Bsa I-HFv2 (#R3733L, NEB) and 0.5 µL T4 DNA Ligase (#M0202M, NEB) in T4 DNA ligase buffer. The reaction was incubated in a thermocycler for 10 cycles of 10 min at 37°C and 10 min at 16°C, then heated to 50°C for 5 min and then 80°C for 5 min. 2 µL of ligation mixture was used to transform *Escherichia coli* competent cells. The assembled plasmids were identified using PCR and further confirmed by Sanger sequencing. PCR primers and sequencing primers are listed in Table S4. Sequences of DdCBE plasmids constructed in this study are list in Supplementary sequence.

Cell culture and nucleofection

HEK293FT cells (Thermofisher, R70007) were cultured in DMEM supplemented with 10% FBS at 37°C with 5% CO_2, and detected without mycoplasma contamination by PCR test. Cells were co-transfected with 50 ng pEGFP-N1 containing the target fragment, 300 ng left-DdCBE and 300 ng right-DdCBE using lonza 4D-Nucleofector by SF Cell Line 4D-Nucleofector X Kit. Cells were supplemented with 2 μg/mL puromycin 24 hrs post nucleofection, and collected at day 3 and day 6 for DNA extraction.

In vitro transcription of DdCBE mRNA

The mRNA of DdCBE pair was transcribed with mMESSAGE mMACHINE T7 Ultra Kit (Life, AM1345) according to the manufacturer's protocol. In brief, equal molar of left-MTS-DdCBE and right-MTS-DdCBE containing T7 promoter were mixed and linearized with Pme I (NEB). Then the linearized DNA was purified with DNA Clean & ConcentratorTM-5 (Zymo Research). 500 ng of purified DNA was used for transcription. The transcribed mRNA was recovered by the RNA Clean & ConcentratorTM-5 (Zymo Research) and stored at -80°C until use.

Zebrafish husbandry

All animal experimentations were carried out in accordance with approved guidelines of the Institutional Animal Care and Use Committee of the Nanjing University. All zebrafish lines were kept on AB background. Adult zebrafish were maintained with automatic fish housing system at 28°C under a 14/10 hrs light-dark cycle. Zebrafish embryos were maintained at 28°C in 10% Hank's solution and staged with standard techniques.

Micro-injection of one-cell stage zebrafish embryos

Transcribed mRNA and phenol red were co-injected into one-cell stage fertilized zebrafish eggs as previous described [4]. Each embryo was injected with 1 nL of solution then incubated at 28°C for the following experiments.

Zebrafish swimming assay

A swim-tracking test was performed as previously described [5]. Zebrafish were placed in an opaque acrylic tank (length: 11.5 cm; width: 9 cm) containing aquarium water (depth: 6 cm) and acclimatized for 2 min. Zebrafish swimming was recorded for 5 or 10 min with Ethovision XT software (Noldus, Wageningen, Netherlands). At least eight zebrafish for each group were analyzed for this assay. Uninjured and sham-injured zebrafish were used as controls.

Transmission electron microscopy

Zebrafish muscle tissues were dissected and fixed with 2.5% (v/v) glutaraldehyde in 0.2 M cacodylate buffer (50 mM cacodylate, 50 mM KCl, and 2.5 mM MgCl2, pH 7.2) overnight. After washing with cacodylate buffer, the tissues were cut into approximately 1-2 mm3 pieces and immersed in 1% OsO_4 in 0.2 M cacodylate buffer for 2 hrs at 4°C. Then the samples were washed and submerged in 0.5% uranyl acetate overnight, dehydrated through a graded series of ethanol, and embedded in resin (Low Viscosity Embedding Media Spurr's Kit, EMS, 14300). Ultrathin sections were cut on an ultramicrotome and mounted on copper grids. The sections were stained with uranyl acetate and lead citrate and observed using a JEM-1400 transmission electron microscope (JEOL).

DNA extraction and genotyping

Genomic DNA of HEK293FT cells were extracted with QuickExtract™ DNA Extraction Solution (Lucigen). Live zebrafish embryo genotyping was performed as previous described[6]. Briefly, at day 3 post-fertilization, single fish embryo was put into one well of 96-well plate filled with DNA collection buffer (with proteinase K 15 μg/mL) and shaken for 30 min at 37°C, then 5 μL solution containing skin cell was used as PCR template. Genomic DNAs of zebrafish embryo, juvenile zebrafish and adult zebrafish fish organs were prepared using DNA collection buffer with proteinase K (15 μg/mL) in 0.3M Tirs-HCl pH8.0 buffer. The fragments spanning the editing sites were amplified with specific primers for Sanger sequencing. Primers are listed in the Table S4.

Western blot for DdCBE expression

Injected embryos were collected at 12 hpf, 24 hpf and 48 hpf, and homogenized by lysis buffer (50 mM Tris-Cl pH7.4, 150 mM NaCl, 1% Triton X-100, 0.1% SDS and protease inhibitors cocktail). 50 μg protein of each sample was loaded onto 10% gels for electrophoresis, and then transferred to a PVDF membrane (Millipore). Membranes were incubated with primary antibody overnight at 4°C using anti-GAPDH (Santa Cruz, sc-32233) and Anti-DDDDK-tag pAb (MBL, PM020), followed by wash with TBST and incubation with second antibodies (peroxidase Goat anti-Rabbit or Goat anti-mouse IgG) for 1 h at room temperature. Signal was detected with enhanced chemiluminescence detection reagent (Vazyme) and imaged by Tannon4500 SF.

Immunofluorescent staining

HEK293FT cells were nucleofected with DdCBE pair, and labelled with 100 nM Mito-Tracker (Beyotime, C1035) for 30 min at 37°C with 5% CO_2 48 hrs post-transfection. Then

cells were fixed with 4% PFA and penetrated with 0.05% Triton X-100. After blocking with 5% BSA for 2 hrs at room temperature, the cells were incubated with anti-DDDDK-tag and Alexa Flour-conjugated anti-rabbit IgG sequentially. Images were captured using Laser scanning confocal microscope (ZEISS LSM800).

Deep sequencing

Genomic regions of interest were firstly amplified with barcoded primers (first round PCR, PCR1) using Phanta Max Super-Fidelity DNA Polymerase (Vazyme). The PCR1 products were pooled with equal moles and purified for the second round PCR (PCR2). PCR2 was performed using index primers (Vazyme) and purified by DNA Clean Beads for sequencing using Illumina NovaSeq platform. Barcoded primers used for PCR1 are listed in Table S4.

Whole mtDNA sequencing

Whole mtDNA sequencing was performed as previously reported [3]. In brief, two overlapping fragments around 8 kb each were amplified and purified using gel extraction. The two fragments were pooled with equal moles and subjected to library preparation using TruePrepTM DNA Library Prep Kit V2 for Illumina (Vazyme). The libraries were purified using DNA Clean Beads by 0.5×/0.35× double size selection. Libraries were pooled and sequenced by Illumina NovaSeq platform. Primers for amplification of long-range PCR are listed in Table S4.

To detect SNPs in signal embryos of our wild-type strain, we used ATAC-seq to perform whole mitochondrial genome sequencing as previously described [3]. In brief, single

zebrafish embryos were tagmented with 0.5 μL Tn5 transposase (Vazyme) in a total volume of 10 μL lysis buffer containing 1×TD buffer, 0.2% NP-40 and 0.3×PBS. Reactions were incubated at 37°C for 30 min on a thermomixer and stored at -80°C until use. Libraries were prepared using TruePrepTM DNA Library Prep Kit V2 for Illumina (Vazyme). 200-1000 bp were selected using Fast Pure Gel DNA Extraction Mini Kit (Vazyme). Libraries were pooled and sequenced by Illumina NovaSeq platform.

Deep sequencing data analysis

The Danio rerio (zebrafish) mitochondrial genome reference sequence (NC_002333) was downloaded from NCBI database. Bowtie2 was used to build the alignment index using default parameters. Paired end reads with overlap were merged into a single read, and bowtie2 was used for alignment in single end mode. Otherwise, reads were mapped in paired end mode by using bowtie2 with default parameters. Alignment results were converted to bam format using samtools and visualized in Integrative Genomics Viewer (IGV). Bases with depth over 2 million were truncated to 2 million, and only C-to-T or G-to-A conversion was calculated for DdCBE-mediated editing.

Whole mtDNA sequencing data analysis

For sequencing data, quality control by fastqc and trim_galore in paired end mode was performed. The Illumina adapter sequence or Ns in either side of the read was trimmed, and only reads with quality over 20 were kept for further analysis. QC-passed reads were mapped to NC_002333 by using bowtie2 with default parameters of paired end. The DdCBE editing efficiency was calculated as mentioned above.

Off-target analysis

SNP sites of zebrafish were obtained from Variation VCF in Ensembl database, which includes 64 annotated C/G to T/A variation, and from SNPFisher database [7], which includes 66 annotated C/G to T/A variation. The combined SNP dataset was used for further analysis. For the off-target analysis, the following sites were excluded before analysis and visualization: (1) the above obtained SNP sites; (2) the sites of which C/G to T/A variation over 1% in any control sample; (3) the evident SNP sites of which C/G to T/A variation over 90% in any sample; (4) sites within the DdCBE spacing region. The average off-target editing frequency was calculated as described before [3] . Briefly, the sites with C/G to T/A conversion were used for calculating average off-target editing frequency independently for each biological replicate as: the sum events of C/G to T/A conversion were divided by the total coverage of these sites.

Reference

1 Huang, P. *et al.* Heritable gene targeting in zebrafish using customized TALENs. *Nat Biotechnol* **29**, 699-700 (2011).

2 Cermak, T. *et al.* Efficient design and assembly of custom TALEN and other TAL effector-based constructs for DNA targeting. *Nucleic Acids Res* **39**, e82 (2011).

3 Mok, B. Y. *et al.* A bacterial cytidine deaminase toxin enables CRISPR-free mitochondrial base editing. *Nature* **583**, 631-637 (2020).

4 Suster, M. L., Kikuta, H., Urasaki, A., Asakawa, K. & Kawakami, K. Transgenesis in zebrafish with the tol2 transposon system. *Methods Mol Biol* **561**, 41-63 (2009).

5 Fang, P. *et al.* HMGB1 contributes to regeneration after spinal cord injury in adult zebrafish. *Mol Neurobiol* **49**, 472-483 (2014).

6 Zhang, X., Zhang, Z., Zhao, Q. & Lou, X. Rapid and Efficient Live Zebrafish Embryo Genotyping. *Zebrafish* **17**, 56-58 (2020).

7 Butler, M. G. *et al.* SNPfisher: tools for probing genetic variation in laboratory-reared zebrafish. *Development* **142**, 1542-1552 (2015).

Figure S1

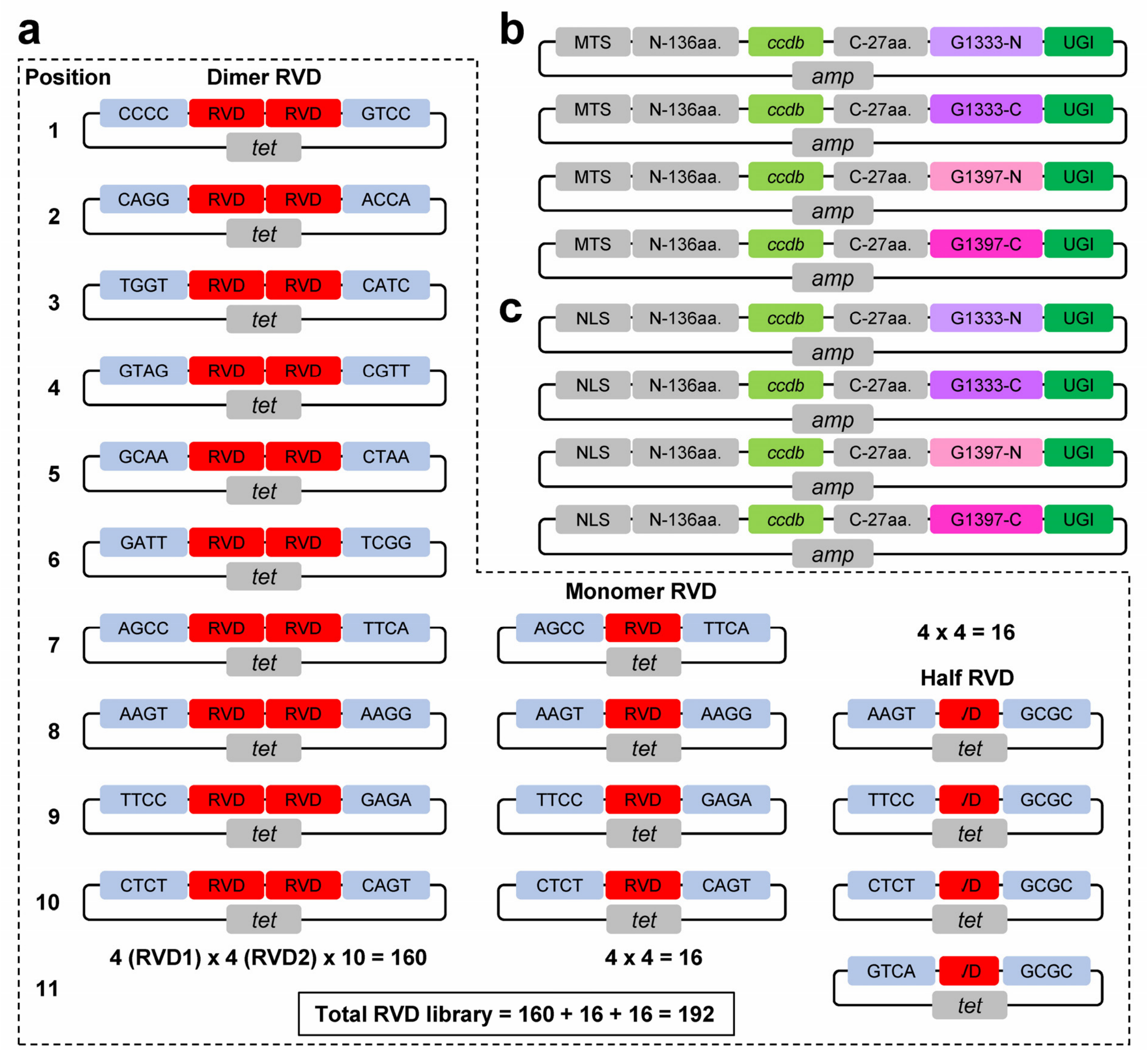

Figure S1. Architecture of RVD library and half DdCBE backbones.

(a) Scheme of RVD plasmid library. A total of 192 RVD plasmids were constructed including 160 dimer RVD modules, 16 monomer RVD modules and 16 half RVD modules. (b, c) Scheme of DdCBE backbones with half $DddA_{tox}$ and mitochondrial localization signal (b) or nuclear localization signal (c).

Figure S2

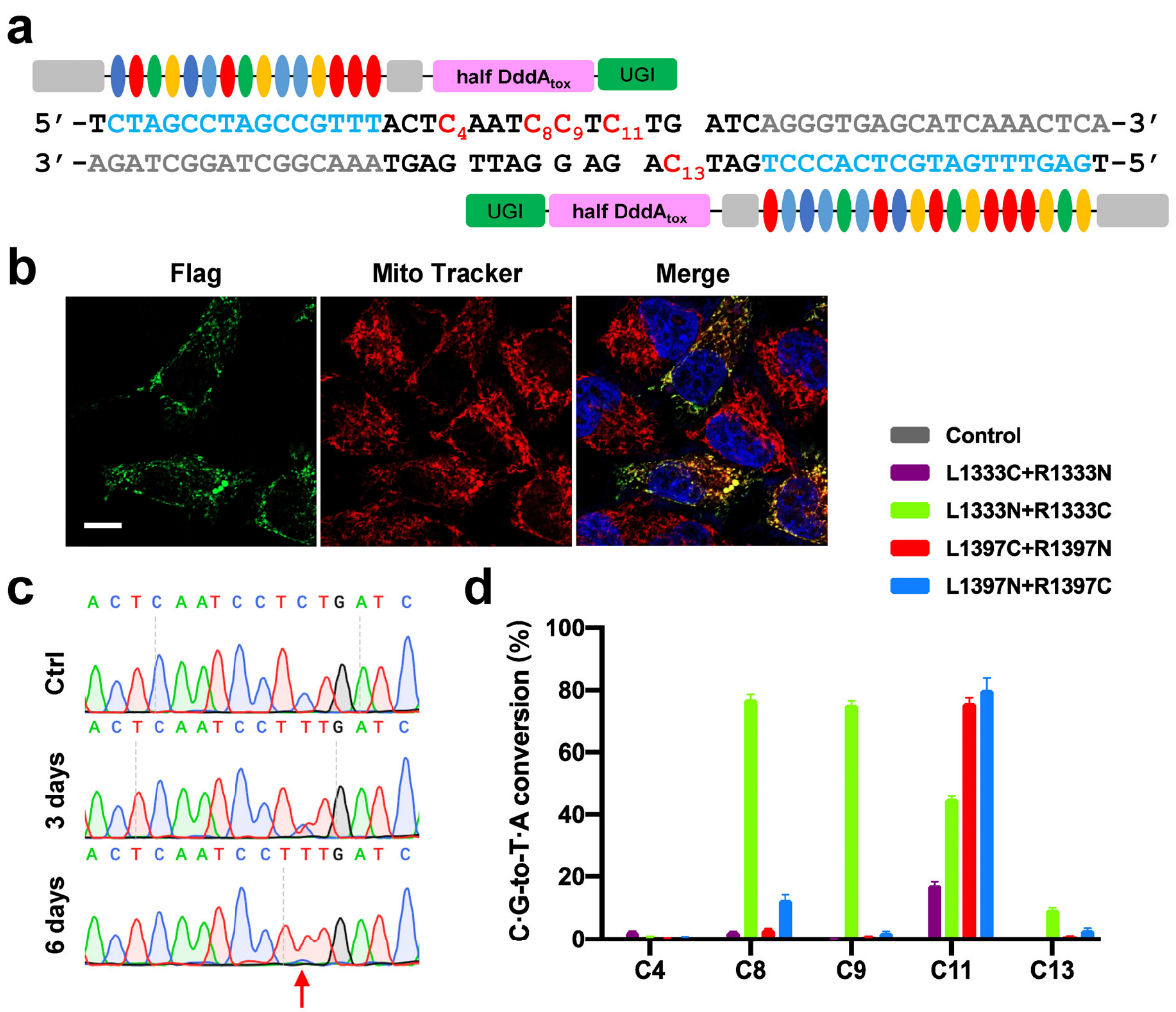

Figure S2. Activity of one-step assembled DdCBE pair in HEK293FT cells.

(a) The assembled DdCBE pairs enable targeting *MT-ND1*. Cytosine in spacing region are numbered and shown in red. RVDs recognition sequences are in blue.

(b) Fluorescence imaging of flag-tagged DdCBE in HEK293FT cells. Mitochondria were labeled with Mito Tracker. Scale bar, 25 μm.

(c) Confirmation of editing in the spacing region by Sanger sequencing at day 3 and 6 post transfection. The untreated cells were used as control. The targeted site is indicated by the red arrow.

(d) Editing efficiencies of DdCBE pairs determined by deep sequencing. The data are presented as Means ± SEM (n = 3).

Figure S3

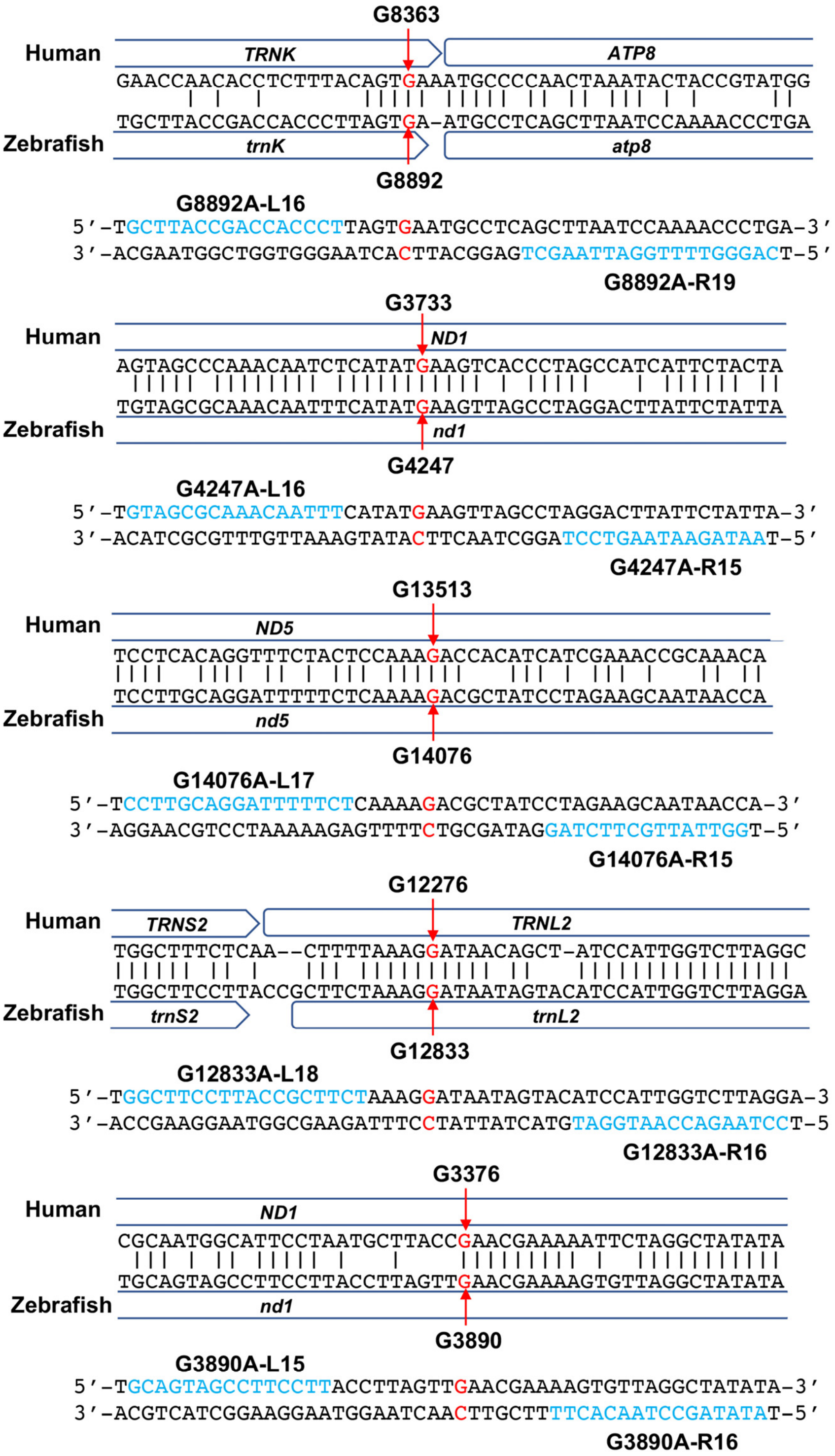

Figure S3. Conservation analysis of targeting sites between human and zebrafish. The target base is shown in red and indicated by arrow. RVDs recognition sequences are in blue.

Figure S4

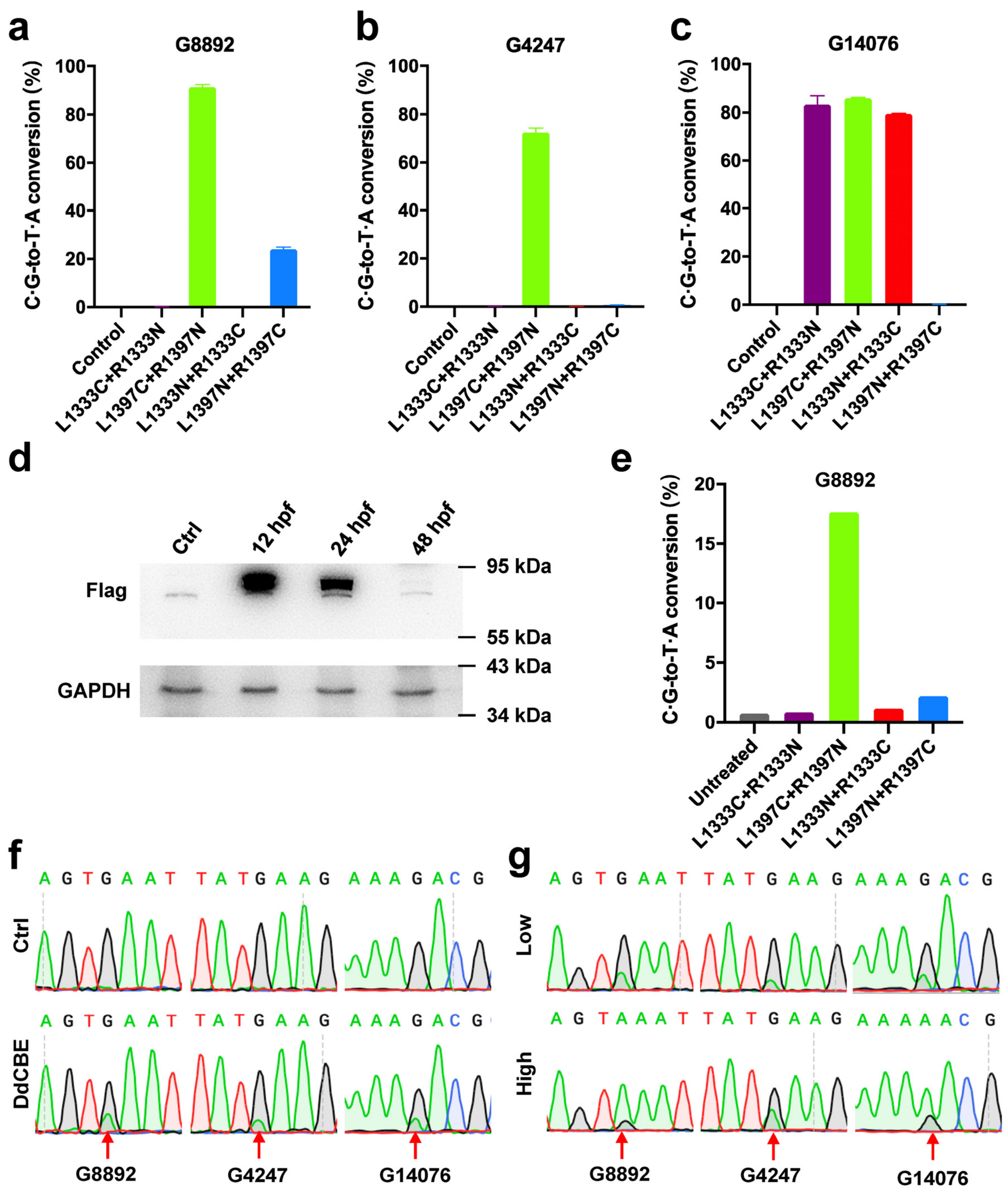

Figure S4. DdCBE-mediated mtDNA mutations in zebrafish.

(a-c) The editing efficiencies of NLS-DdCBE pairs targeting G8892 (a), G4247 (b) and G14076 (c) in HEK293FT cells.

(d) Expression of MTS-DdCBE pair (L1397C + R1397N) in zebrafish embryos injected with

DdCBE mRNA at 12, 24 and 48 hpf. Untreated zebrafish embryos were used as control.
(e) The activity of MTS-DdCBE pairs targeting G8892 in pooled embryos.
(f) Confirmation of mtDNA editing by Sanger sequencing in pooled embryos treated with DdCBE pair's mRNA for G8892, G4247 and G14076.
(g) Representative sequence chromatograms of G8892, G4247, and G14076 founders with low and high editing efficiency. The target sites are indicated by the red arrow.

Figure S5

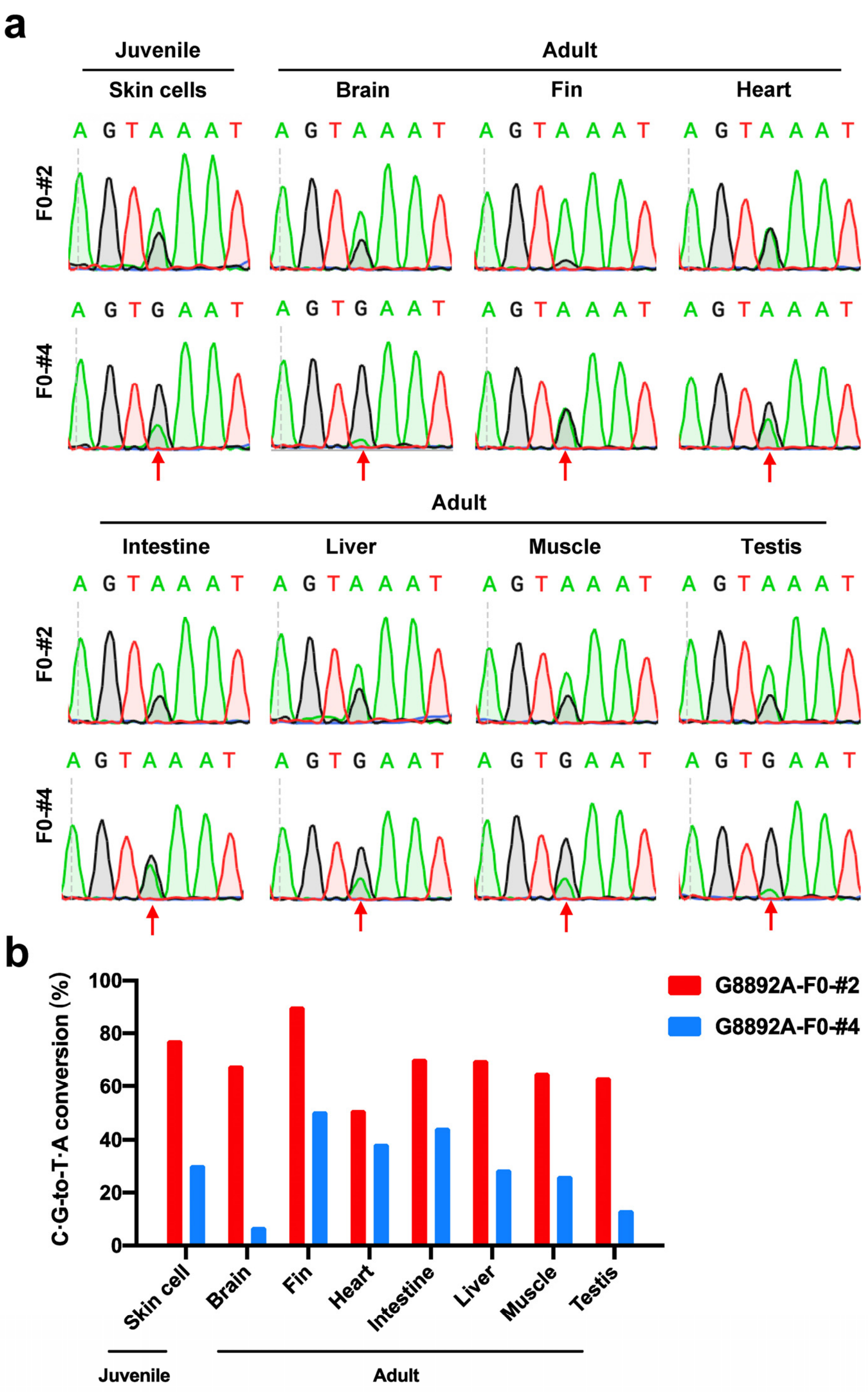

Figure S5. DdCBE-mediated editing in tissues from founders.

(a) Sequence chromatograms of tissues in G8892 #2 and #4 founders. The edited site are indicated by arrows.

(b) Deep sequencing analysis of C·G-to-T·A conversions in G8892 #2 and #4 founders.

Figure S6

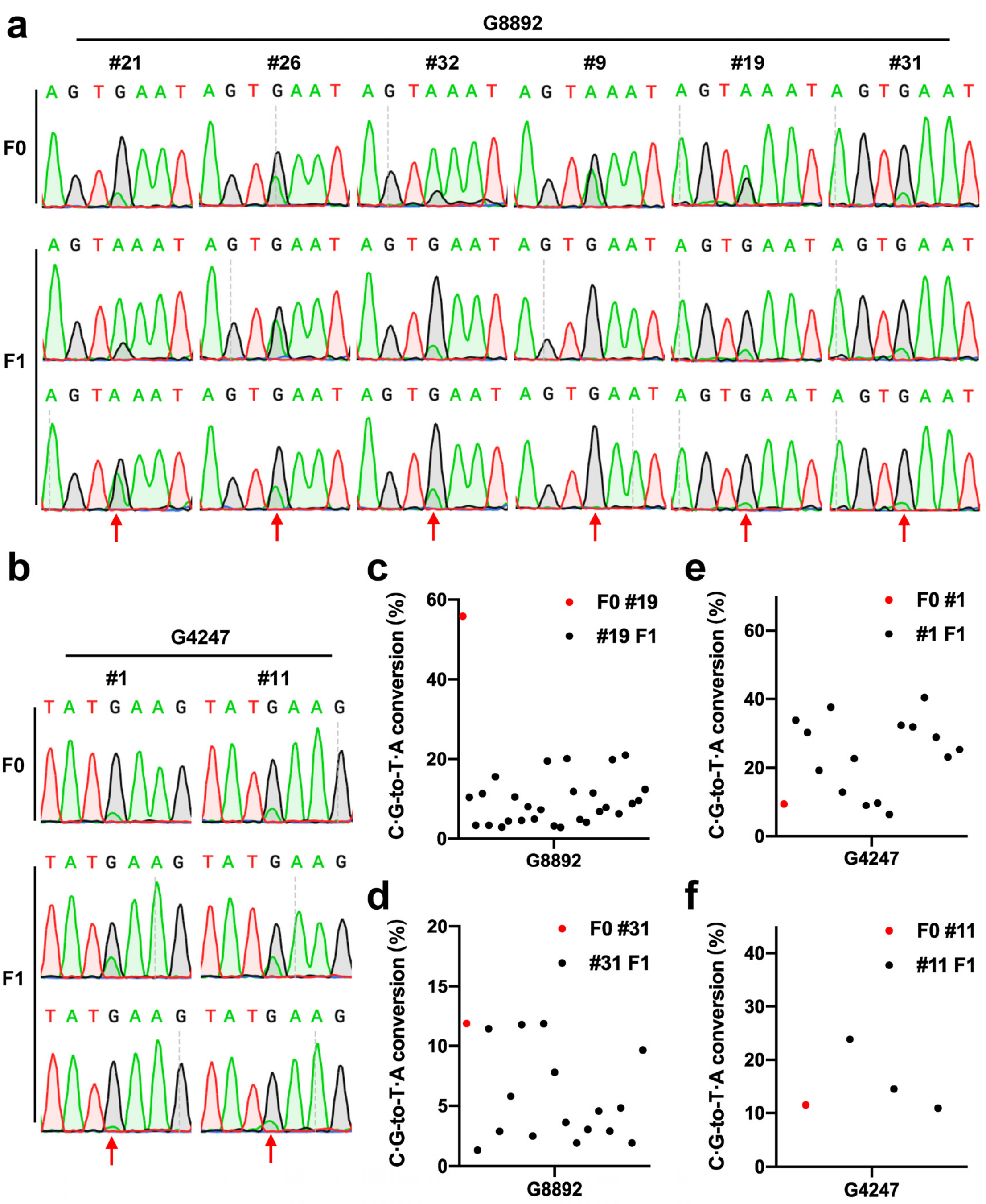

Figure S6. Germline transmission of mtDNA base editing.

(a, b) Representative sequence chromatograms of mtDNA editing in G8892 F0 (a), G4247 F0 (b) and their corresponding F1.

(c-f) Deep sequencing analysis of transmission of mtDNA mutation in founders and their corresponding offspring. Red and black dots indicate F0 and F1, respectively.

Figure S7

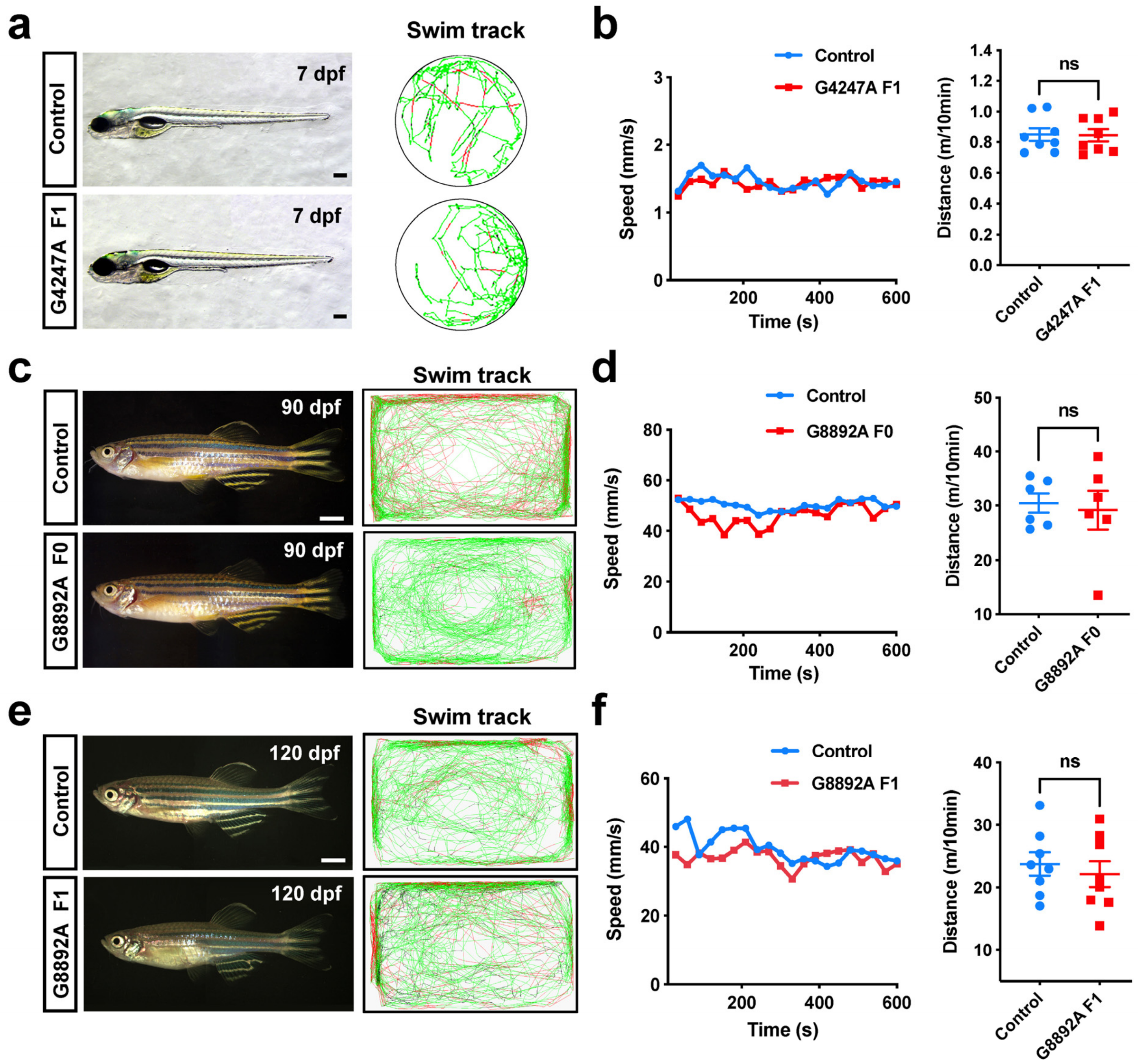

Figure S7. Morphology feature and behavior analysis of mtDNA edited fish.

(a) Left panels, live images of control and G4247A F1 zebrafish larvae at 7 days post-fertilization. Lateral view, anterior to the left. Scale bar, 20 μm. Right panels, representative swim tracking of individual animals. Colour coding represents movement speed: red, faster than 75 mm/second; green, 25-75 mm/s; black, slower than 25 mm/s. (b) Quantification of total distance and speed of G4247A F1 zebrafish at 7 days post-fertilization (Means ± SEM, n = 12 for each group). Significance was calculated with unpaired two-tailed Student's t test (ns, not significant).

(c) Left panels, live images of control and G8892A F0 zebrafish at 90 days post-fertilization. Lateral view, anterior to the left. Scale bar, 20 mm. Right panels, representative swim tracking of individual animals.
(d) Quantification of total distance and speed of G8892A F0 zebrafish at 90 days post-fertilization (Means ± SEM, n = 6 for each group). Significance was calculated with unpaired two-tailed Student's t test (ns, not significant).
(e) Left panels, live images of control and G8892A F1 zebrafish at 120 days post-fertilization. Scale bar, 2 mm. Right panels, representative swim tracking of individual animals.
(f) Quantification of total distance and speed of G8892A F1 zebrafish at 120 days post-fertilization (Means ± SEM, n = 8 for each group). Significance was calculated with unpaired two-tailed Student's t test (ns, not significant).

Figure S8

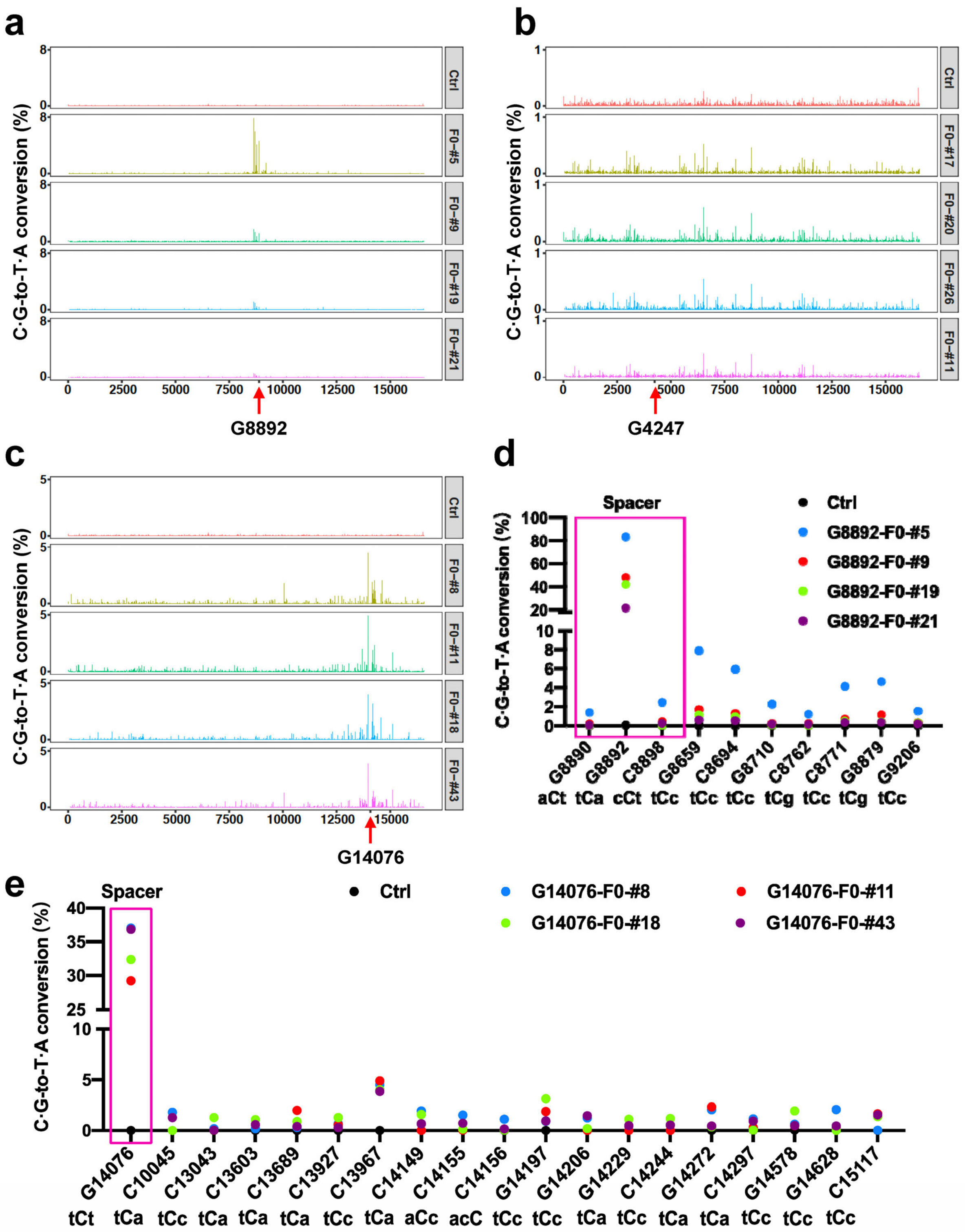

Figure S8. Off-target analysis by whole mtDNA sequencing.

(a-c) Whole mtDNA sequencing analysis of off-target sites in G8892 (a), G4247 (b) and G14076 (c) founders. The maximum C·G-to-T·A conversion rate at each site from 5 wild-type fish was selected as a representative control, which was used in a-c panels.

(d, e) Off-target sites with conversion rate over 1% in any sample were displayed in G8892 (d), and G14076 (e) founders. Spacing region is boxed in red. Motifs of off-target sites are labelled at the bottom.

Figure S9

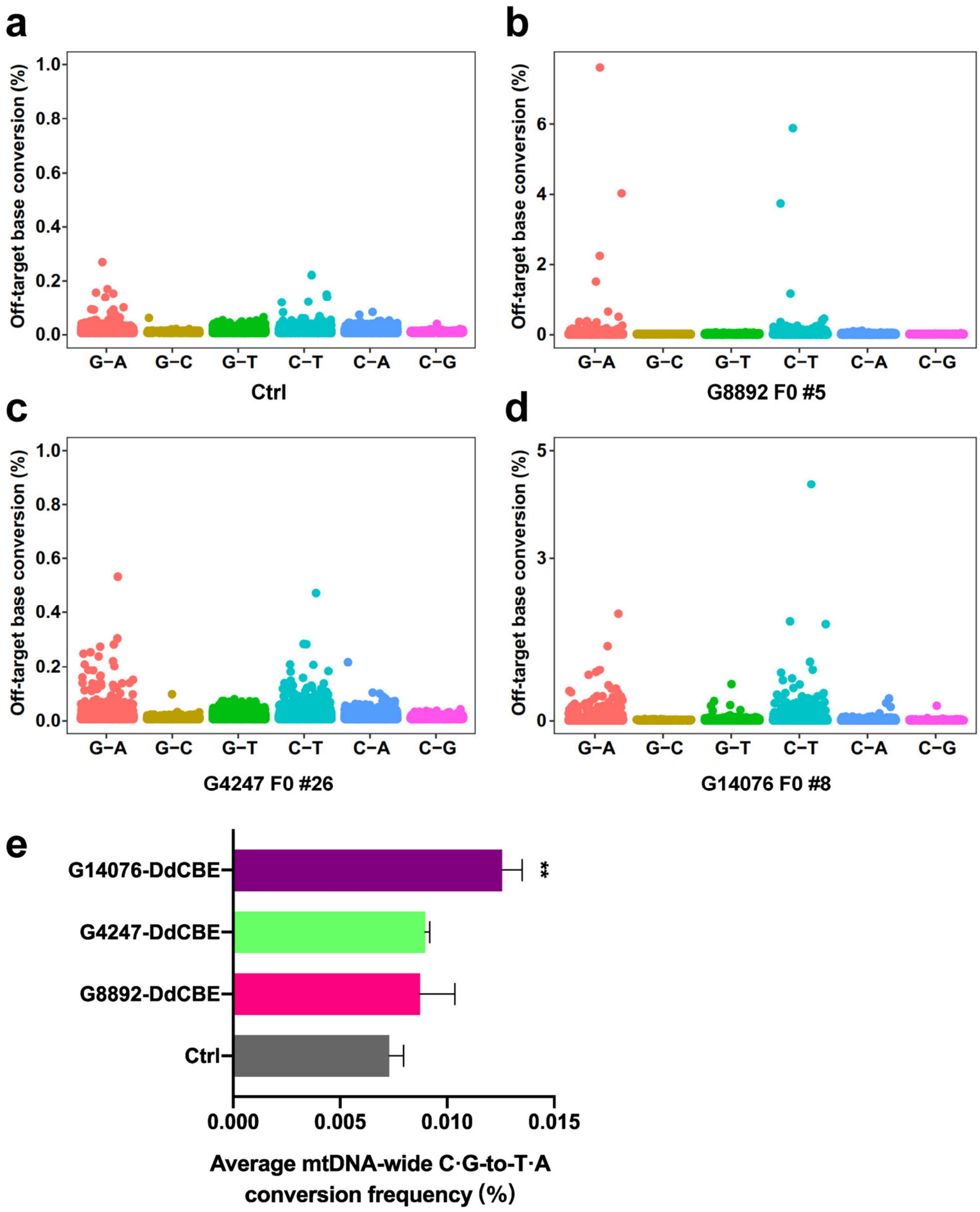

Figure S9. Off-target analysis in founders.

(a-d) The frequency of off-target base conversions in control (a), G8892 F0 #5 (b), G4247 F0 #26 (c) and G14076 F0 #8 (d).

(e) Average frequency of mtDNA-wide C·G-to-T·A off-target conversions for each DdCBE pair and control. Values and error bars reflect Means ± SEM (n = 4 for each DdCBE treated group, n = 5 for control). Significance was calculated with unpaired two-tailed Student’s t test (**, $P < 0.01$).

Table S1. Summary of DdCBE toxicity on zebrafish embryos.

Substance	Injection dosage (pg)	Replicates	Total # of injected embryos	24 hrs SR (%)	SD	96 hrs SR (%)	SD	Nonspecific toxicity (%)
Phenol red	—	3	314	93.7	7.6	93.8	3.6	—
G8892	50	3	309	94.9	7.8	94	7.7	5.8
	200	3	372	90.1	4.9	89.3	5.6	13.4
G4247	50	3	345	92	8.4	90.6	6.6	6.7
	200	3	348	87.5	9.6	81	8.2	21

SR, survival rates. SD, standard deviation. Nonspecific toxicity determined by the portion of live embryos exhibit defects or developmental delay in all replicates.

Table S2. Summary of mtDNA base editing efficiency of founders.

Site	Tested individuals	Edited individuals	Mutation load
G8892	80	66	5.78% -88.32%
G4247	30	8	6.36% -23.48%
G14076	72	43	9.31% -67.9%

Table S3. Summary of germline transmission in offspring.

Site	Founder fish	F0 mutation load in skin cells	Edited F1/Total	Mutation load
G8892	#9	46.02%	0/40	0
	#19	55.80%	28/40	2.82% -20.97%
	#21	16.71%	38/80	50.95% -84.33%
	#26	46.39%	30/48	4.95% -58.54%
	#31	11.90%	16/40	1.33% -11.88%
	#32	88.32%	29/40	5.59% -30.54%
G4247	#1	9.46%	20/40	6.32% -40.42%
	#11	12.20%	4/40	10.87% -23.83%

Table S4. Primers information.

	Primers	Sequence (5'-3')
Primers for RVD	RVD seq For	TGACCGCAGTGGAGGCAGTG
	RVD seq Rev	TTCACTGCATCCAGCGCAGG
Primers for genotyping	zeb-G3890A-seq-Fwd	AAGGATCGGAAAAAGGGGGC
	zeb-G3890A-seq-Rev	AGTCCTCGGGGGCCTATTAC
	zeb-G4247A-seq-Fwd	ACCCATACCCATGCCCTATC
	zeb-G4247A-seq-Rev	CCCCCTCTGTAAGATCGAACG
	zeb-G8892A-seq-Fwd	CCCATTGTAGTCGAAGCCGT
	zeb-G8892A-seq-Rev	CCTAAAAGGTAGGGGCTCGC
	zeb-G12833A-seq-Fwd	TAGGGGTTAAAGCCCCCTCA
	zeb-G12833A-seq-Rev	GACCAGGTGATGAATAAGGCGA
	zeb-G14076-seq-Fwd	GGGGAACTTACCACACCCTAC
	zeb-G14076-seq-Rev	TGCGGTAAATGATGTGGCGA
Barcoded Primers for PCR1	zeb-G4247A-i5-Fwd1	ACACTCTTTCCCTACACGACGCTCTTCCGATCTCAA CCTGGGATCAGGTTGAGCAT
	zeb-G4247A-i5-Fwd2	ACACTCTTTCCCTACACGACGCTCTTCCGATCTTCA CCTGGGATCAGGTTGAGCAT
	zeb-G4247A-i5-Fwd3	ACACTCTTTCCCTACACGACGCTCTTCCGATCTACC CCTGGGATCAGGTTGAGCAT
	zeb-G4247A-i5-Fwd4	ACACTCTTTCCCTACACGACGCTCTTCCGATCTGTA CCTGGGATCAGGTTGAGCAT
	zeb-G4247A-i5-Fwd5	ACACTCTTTCCCTACACGACGCTCTTCCGATCTCAG CCTGGGATCAGGTTGAGCAT
	zeb-G4247A-i7-Rev	GTGACTGGAGTTCAGACGTGTGCTCTTCCGATCTT GGTCCTGCTGCATACTCTAC
	zeb-G8892A-i5-Fwd1	ACACTCTTTCCCTACACGACGCTCTTCCGATCTCAA TAGAAGACGCCTCACTAAGATGC
	zeb-G8892A-i5-Fwd2	ACACTCTTTCCCTACACGACGCTCTTCCGATCTTCA TAGAAGACGCCTCACTAAGATGC
	zeb-G8892A-i5-Fwd3	ACACTCTTTCCCTACACGACGCTCTTCCGATCTACC TAGAAGACGCCTCACTAAGATGC
	zeb-G8892A-i5-Fwd4	ACACTCTTTCCCTACACGACGCTCTTCCGATCTGTA TAGAAGACGCCTCACTAAGATGC
	zeb-G8892A-i5-Fwd5	ACACTCTTTCCCTACACGACGCTCTTCCGATCTCAG TAGAAGACGCCTCACTAAGATGC
	zeb-G8892A-i7-Rev	GTGACTGGAGTTCAGACGTGTGCTCTTCCGATCTG TAGGGGCTCGCGAATTGAT
	zeb-G14076A-i5-Fwd1	ACACTCTTTCCCTACACGACGCTCTTCCGATCTCAA GGGGAACTTACCACACCCTAC
	zeb-G14076A-i5-Fwd2	ACACTCTTTCCCTACACGACGCTCTTCCGATCTTCA GGGGAACTTACCACACCCTAC
	zeb-G14076A-i5-Fwd3	ACACTCTTTCCCTACACGACGCTCTTCCGATCTACC GGGGAACTTACCACACCCTAC
	zeb-G14076A-i5-Fwd4	ACACTCTTTCCCTACACGACGCTCTTCCGATCTGTA GGGGAACTTACCACACCCTAC
	zeb-G14076A-i5-Fwd5	ACACTCTTTCCCTACACGACGCTCTTCCGATCTCAG GGGGAACTTACCACACCCTAC
	zeb-G14076A-i7-Rev	GTGACTGGAGTTCAGACGTGTGCTCTTCCGATCTT GCGGTAAATGATGTGGCGA

	EGPP-T-i5-Fwd1	ACACTCTTTCCCTACACGACGCTCTTCCGATCTCAA GATCACTCTCGGCATGGACG
	EGPP-T-i5-Fwd2	ACACTCTTTCCCTACACGACGCTCTTCCGATCTTCA GATCACTCTCGGCATGGACG
	EGPP-T-i5-Fwd3	ACACTCTTTCCCTACACGACGCTCTTCCGATCTACC GATCACTCTCGGCATGGACG
	EGPP-T-i5-Fwd4	ACACTCTTTCCCTACACGACGCTCTTCCGATCTGTA GATCACTCTCGGCATGGACG
	EGPP-T-i5-Fwd5	ACACTCTTTCCCTACACGACGCTCTTCCGATCTCAG GATCACTCTCGGCATGGACG
	EGPP-T-i7-Rev	GTGACTGGAGTTCAGACGTGTGCTCTTCCGATCTA CAAACCACAACTAGAATGCAGTG
**Primers for long-range PCR**	zeb-mt F1-Fwd	GAAAGGGAAGGAACTCGGCAAAC
	zeb-mt F1-Rev	AGAAGAGGTGTTGATAGTGGGTCTG
	zeb-mt F2-Fwd	CCTAGCACTCCTAGTAGCCACAG
	zeb-mt F2-Rev	CTCCAAAGGGTCTTCTCGTCTTGTA

Supplementary sequence

DdCBE domains are annotated as: red for MTS, italics for linker, yellow for flag tag, green for N&C-terminal domain, underlined for RVD, purple for half of $DddA_{tox}$, cyan for UGI. All amino acid sequences of DdCBE vectors targeting G4247A are displayed. Only RVDs sequences are showed for G3890A, G8892A, G12833A and G14076A.

Zebrafish G4247A Left TALE-G1333C-UGI

MLGFVGRVAAAPASGALRRLTPSASLPPAQLLLRAAPTAVHPVRDYAAQTSESGGGGSPG*AAA*D
YKDDDDK*GS*VDLRTLGYSQQQQEKIKPKVRSTVAQHHEALVGHGFTHAHIVALSQHPAALGTVA
VKYQDMIAALPEATHEAIVGVGKQWSGARALEALLTVAGELRGPPLQLDTGQLLKIAKRGGVTA
VEAVHAWRNALTGAPLNLTPEQVVAIASNNGGKQALETVQRLLPVLCQAHGLTPEQVVAIASNG
GGKQALETVQRLLPVLCQAHGLTPEQVVAIASNIGGKQALETVQRLLPVLCQAHGLTPDQVVAI
ASNNGGKQALETVQRLLPVLCQAHGLTPEQVVAIASHDGGKQALETVQRLLPVLCQAHGLTPAQ
VVAIASNNGGKQALETVQRLLPVLCQAHGLTPEQVVAIASHDGGKQALETVQRLLPVLCQAHGL
TPAQVVAIASNIGGKQALETVQRLLPVLCQAHGLTPDQVVAIASNIGGKQALETVQRLLPVLCQ
AHGLTPDQVVAIASNIGGKQALETVQRLLPVLCQAHGLTPDQVVAIASHDGGKQALETVQRLLP
VLCQAHGLTPAQVVAIASNIGGKQALETVQRLLPVLCQAHGLTPDQVVAIASNIGGKQALETVQ
RLLPVLCQAHGLTPDQVVAIASNGGGKQALETVQRLLPVLCQAHGLTPEQVVAIASNGGGKQAL
ETVQRLLPVLCQAHGLTPEQVVAIASNGGGRPALESIVAQLSRPDPALAALTNDHLVALACLGG
RPALDAVKKGLG*GS*PTPYPNYANAGHVEGQSALFMRDNGISEGLVFHNNPEGTCGFCVNMTETL
LPENAKMTVVPPEGAIPVKRGATGETKVFTGNSNSPKSPTKGGC*SGGS*TNLSDIIEKETGKQLV
IQESILMLPEEVEEVIGNKPESDILVHTAYDESTDENVMLLTSDAPEYKPWALVIQDSNGENKI
KML

Zebrafish G4247A Left TALE-G1397C-UGI

MLGFVGRVAAAPASGALRRLTPSASLPPAQLLLRAAPTAVHPVRDYAAQTSESGGGGSPG*AAA*D
YKDDDDK*GS*VDLRTLGYSQQQQEKIKPKVRSTVAQHHEALVGHGFTHAHIVALSQHPAALGTVA
VKYQDMIAALPEATHEAIVGVGKQWSGARALEALLTVAGELRGPPLQLDTGQLLKIAKRGGVTA
VEAVHAWRNALTGAPLNLTPEQVVAIASNNGGKQALETVQRLLPVLCQAHGLTPEQVVAIASNG
GGKQALETVQRLLPVLCQAHGLTPEQVVAIASNIGGKQALETVQRLLPVLCQAHGLTPDQVVAI
ASNNGGKQALETVQRLLPVLCQAHGLTPEQVVAIASHDGGKQALETVQRLLPVLCQAHGLTPAQ
VVAIASNNGGKQALETVQRLLPVLCQAHGLTPEQVVAIASHDGGKQALETVQRLLPVLCQAHGL
TPAQVVAIASNIGGKQALETVQRLLPVLCQAHGLTPDQVVAIASNIGGKQALETVQRLLPVLCQ
AHGLTPDQVVAIASNIGGKQALETVQRLLPVLCQAHGLTPDQVVAIASHDGGKQALETVQRLLP
VLCQAHGLTPAQVVAIASNIGGKQALETVQRLLPVLCQAHGLTPDQVVAIASNIGGKQALETVQ
RLLPVLCQAHGLTPDQVVAIASNGGGKQALETVQRLLPVLCQAHGLTPEQVVAIASNGGGKQAL
ETVQRLLPVLCQAHGLTPEQVVAIASNGGGRPALESIVAQLSRPDPALAALTNDHLVALACLGG
RPALDAVKKGLG*GS*AIPVKRGATGETKVFTGNSNSPKSPTKGGC*SGGS*TNLSDIIEKETGKQLV

IQESILMLPEEVEEVIGNKPESDILVHTAYDESTDENVMLLTSDAPEYKPWALVIQDSNGENKI
KML

Zebrafish G4247A Left TALE-G1333N-UGI

MLGFVGRVAAAPASGALRRLTPSASLPPAQLLLRAAPTAVHPVRDYAAQTSESGGGGSPG*AAA*D
YKDDDDK*GS*VDLRTLGYSQQQQEKIKPKVRSTVAQHHEALVGHGFTHAHIVALSQHPAALGTVA
VKYQDMIAALPEATHEAIVGVGKQWSGARALEALLTVAGELRGPPLQLDTGQLLKIAKRGGVTA
VEAVHAWRNALTGAPLNLTPEQVVAIASNNGGKQALETVQRLLPVLCQAHGLTPEQVVAIASNG
GGKQALETVQRLLPVLCQAHGLTPEQVVAIASNIGGKQALETVQRLLPVLCQAHGLTPDQVVAI
ASNNGGKQALETVQRLLPVLCQAHGLTPEQVVAIASHDGGKQALETVQRLLPVLCQAHGLTPAQ
VVAIASNNGGKQALETVQRLLPVLCQAHGLTPEQVVAIASHDGGKQALETVQRLLPVLCQAHGL
TPAQVVAIASNIGGKQALETVQRLLPVLCQAHGLTPDQVVAIASNIGGKQALETVQRLLPVLCQ
AHGLTPDQVVAIASNIGGKQALETVQRLLPVLCQAHGLTPDQVVAIASHDGGKQALETVQRLLP
VLCQAHGLTPAQVVAIASNIGGKQALETVQRLLPVLCQAHGLTPDQVVAIASNIGGKQALETVQ
RLLPVLCQAHGLTPDQVVAIASNGGGKQALETVQRLLPVLCQAHGLTPEQVVAIASNGGGKQAL
ETVQRLLPVLCQAHGLTPEQVVAIASNGGGRPALESIVAQLSRPDPALAALTNDHLVALACLGG
RPALDAVKKGLG*GS*GSYALGPYQISAPQLPAYNGQTVGTFYYVNDAGGLESKVFSSGG*SGGS*TN
LSDIIEKETGKQLVIQESILMLPEEVEEVIGNKPESDILVHTAYDESTDENVMLLTSDAPEYKP
WALVIQDSNGENKIKML

Zebrafish G4247A Left TALE-G1397N-UGI

MLGFVGRVAAAPASGALRRLTPSASLPPAQLLLRAAPTAVHPVRDYAAQTSESGGGGSPG*AAA*D
YKDDDDK*GS*VDLRTLGYSQQQQEKIKPKVRSTVAQHHEALVGHGFTHAHIVALSQHPAALGTVA
VKYQDMIAALPEATHEAIVGVGKQWSGARALEALLTVAGELRGPPLQLDTGQLLKIAKRGGVTA
VEAVHAWRNALTGAPLNLTPEQVVAIASNNGGKQALETVQRLLPVLCQAHGLTPEQVVAIASNG
GGKQALETVQRLLPVLCQAHGLTPEQVVAIASNIGGKQALETVQRLLPVLCQAHGLTPDQVVAI
ASNNGGKQALETVQRLLPVLCQAHGLTPEQVVAIASHDGGKQALETVQRLLPVLCQAHGLTPAQ
VVAIASNNGGKQALETVQRLLPVLCQAHGLTPEQVVAIASHDGGKQALETVQRLLPVLCQAHGL
TPAQVVAIASNIGGKQALETVQRLLPVLCQAHGLTPDQVVAIASNIGGKQALETVQRLLPVLCQ
AHGLTPDQVVAIASNIGGKQALETVQRLLPVLCQAHGLTPDQVVAIASHDGGKQALETVQRLLP
VLCQAHGLTPAQVVAIASNIGGKQALETVQRLLPVLCQAHGLTPDQVVAIASNIGGKQALETVQ
RLLPVLCQAHGLTPDQVVAIASNGGGKQALETVQRLLPVLCQAHGLTPEQVVAIASNGGGKQAL
ETVQRLLPVLCQAHGLTPEQVVAIASNGGGRPALESIVAQLSRPDPALAALTNDHLVALACLGG
RPALDAVKKGLG*GS*GSYALGPYQISAPQLPAYNGQTVGTFYYVNDAGGLESKVFSSGGPTPYPN
YANAGHVEGQSALFMRDNGISEGLVFHNNPEGTCGFCVNMTETLLPENAKMTVVPPEG*SGGS*TN
LSDIIEKETGKQLVIQESILMLPEEVEEVIGNKPESDILVHTAYDESTDENVMLLTSDAPEYKP
WALVIQDSNGENKIKML

Zebrafish G4247A Right TALE-G1333C-UGI

MLGFVGRVAAAPASGALRRLTPSASLPPAQLLLRAAPTAVHPVRDYAAQTSESGGGGSPG*AAA*D
YKDDDDK*GS*VDLRTLGYSQQQQEKIKPKVRSTVAQHHEALVGHGFTHAHIVALSQHPAALGTVA
VKYQDMIAALPEATHEAIVGVGKQWSGARALEALLTVAGELRGPPLQLDTGQLLKIAKRGGVTA

VEAVHAWRNALTGAPLNLTPEQVVAIASNIGGKQALETVQRLLPVLCQAHGLTPDQVVAIASNI
GGKQALETVQRLLPVLCQAHGLTPDQVVAIASNGGGKQALETVQRLLPVLCQAHGLTPEQVVAI
ASNIGGKQALETVQRLLPVLCQAHGLTPDQVVAIASNNGGKQALETVQRLLPVLCQAHGLTPEQ
VVAIASNIGGKQALETVQRLLPVLCQAHGLTPDQVVAIASNIGGKQALETVQRLLPVLCQAHGL
TPDQVVAIASNGGGKQALETVQRLLPVLCQAHGLTPEQVVAIASNIGGKQALETVQRLLPVLCQ
AHGLTPDQVVAIASNIGGKQALETVQRLLPVLCQAHGLTPDQVVAIASNNGGKQALETVQRLLP
VLCQAHGLTPEQVVAIASNGGGKQALETVQRLLPVLCQAHGLTPEQVVAIASHDGGKQALETVQ
RLLPVLCQAHGLTPAQVVAIASHDGGKQALETVQRLLPVLCQAHGLTPAQVVAIASNGGGRPAL
ESIVAQLSRPDPALAALTNDHLVALACLGGRPALDAVKKGLG*GS*PTPYPNYANAGHVEGQSALF
MRDNGISEGLVFHNNPEGTCGFCVNMTETLLPENAKMTVVPPEGAIPVKRGATGETKVFTGNSN
SPKSPTKGGC*SGGS*TNLSDIIEKETGKQLVIQESILMLPEEVEEVIGNKPESDILVHTAYDEST
DENVMLLTSDAPEYKPWALVIQDSNGENKIKML

Zebrafish G4247A Right TALE-G1397C-UGI

MLGFVGRVAAAPASGALRRLTPSASLPPAQLLLRAAPTAVHPVRDYAAQTSESGGGGSPG*AAA*D
YKDDDDK*GS*VDLRTLGYSQQQQEKIKPKVRSTVAQHHEALVGHGFTHAHIVALSQHPAALGTVA
VKYQDMIAALPEATHEAIVGVGKQWSGARALEALLTVAGELRGPPLQLDTGQLLKIAKRGGVTA
VEAVHAWRNALTGAPLNLTPEQVVAIASNIGGKQALETVQRLLPVLCQAHGLTPDQVVAIASNI
GGKQALETVQRLLPVLCQAHGLTPDQVVAIASNGGGKQALETVQRLLPVLCQAHGLTPEQVVAI
ASNIGGKQALETVQRLLPVLCQAHGLTPDQVVAIASNNGGKQALETVQRLLPVLCQAHGLTPEQ
VVAIASNIGGKQALETVQRLLPVLCQAHGLTPDQVVAIASNIGGKQALETVQRLLPVLCQAHGL
TPDQVVAIASNGGGKQALETVQRLLPVLCQAHGLTPEQVVAIASNIGGKQALETVQRLLPVLCQ
AHGLTPDQVVAIASNIGGKQALETVQRLLPVLCQAHGLTPDQVVAIASNNGGKQALETVQRLLP
VLCQAHGLTPEQVVAIASNGGGKQALETVQRLLPVLCQAHGLTPEQVVAIASHDGGKQALETVQ
RLLPVLCQAHGLTPAQVVAIASHDGGKQALETVQRLLPVLCQAHGLTPAQVVAIASNGGGRPAL
ESIVAQLSRPDPALAALTNDHLVALACLGGRPALDAVKKGLG*GS*AIPVKRGATGETKVFTGNSN
SPKSPTKGGC*SGGS*TNLSDIIEKETGKQLVIQESILMLPEEVEEVIGNKPESDILVHTAYDEST
DENVMLLTSDAPEYKPWALVIQDSNGENKIKML

Zebrafish G4247A Right TALE-G1333N-UGI

MLGFVGRVAAAPASGALRRLTPSASLPPAQLLLRAAPTAVHPVRDYAAQTSESGGGGSPG*AAA*D
YKDDDDK*GS*VDLRTLGYSQQQQEKIKPKVRSTVAQHHEALVGHGFTHAHIVALSQHPAALGTVA
VKYQDMIAALPEATHEAIVGVGKQWSGARALEALLTVAGELRGPPLQLDTGQLLKIAKRGGVTA
VEAVHAWRNALTGAPLNLTPEQVVAIASNIGGKQALETVQRLLPVLCQAHGLTPDQVVAIASNI
GGKQALETVQRLLPVLCQAHGLTPDQVVAIASNGGGKQALETVQRLLPVLCQAHGLTPEQVVAI
ASNIGGKQALETVQRLLPVLCQAHGLTPDQVVAIASNNGGKQALETVQRLLPVLCQAHGLTPEQ
VVAIASNIGGKQALETVQRLLPVLCQAHGLTPDQVVAIASNIGGKQALETVQRLLPVLCQAHGL
TPDQVVAIASNGGGKQALETVQRLLPVLCQAHGLTPEQVVAIASNIGGKQALETVQRLLPVLCQ
AHGLTPDQVVAIASNIGGKQALETVQRLLPVLCQAHGLTPDQVVAIASNNGGKQALETVQRLLP
VLCQAHGLTPEQVVAIASNGGGKQALETVQRLLPVLCQAHGLTPEQVVAIASHDGGKQALETVQ
RLLPVLCQAHGLTPAQVVAIASHDGGKQALETVQRLLPVLCQAHGLTPAQVVAIASNGGGRPAL
ESIVAQLSRPDPALAALTNDHLVALACLGGRPALDAVKKGLG*GS*GSYALGPYQISAPQLPAYNG

QTVGTFYYVNDAGGLESKVFSSGG*SGGS*TNLSDIIEKETGKQLVIQESILMLPEEVEEVIGNKP
ESDILVHTAYDESTDENVMLLTSDAPEYKPWALVIQDSNGENKIKML

Zebrafish G4247A Right TALE-G1397N-UGI

MLGFVGRVAAAPASGALRRLTPSASLPPAQLLLRAAPTAVHPVRDYAAQTSESGGGGSPG*AAA*D
YKDDDDK*GS*VDLRTLGYSQQQQEKIKPKVRSTVAQHHEALVGHGFTHAHIVALSQHPAALGTVA
VKYQDMIAALPEATHEAIVGVGKQWSGARALEALLTVAGELRGPPLQLDTGQLLKIAKRGGVTA
VEAVHAWRNALTGAPLNLTPEQVVAIASNIGGKQALETVQRLLPVLCQAHGLTPDQVVAIASNI
GGKQALETVQRLLPVLCQAHGLTPDQVVAIASNGGGKQALETVQRLLPVLCQAHGLTPEQVVAI
ASNIGGKQALETVQRLLPVLCQAHGLTPDQVVAIASNNGGKQALETVQRLLPVLCQAHGLTPEQ
VVAIASNIGGKQALETVQRLLPVLCQAHGLTPDQVVAIASNIGGKQALETVQRLLPVLCQAHGL
TPDQVVAIASNGGGKQALETVQRLLPVLCQAHGLTPEQVVAIASNIGGKQALETVQRLLPVLCQ
AHGLTPDQVVAIASNIGGKQALETVQRLLPVLCQAHGLTPDQVVAIASNNGGKQALETVQRLLP
VLCQAHGLTPEQVVAIASNGGGKQALETVQRLLPVLCQAHGLTPEQVVAIASHDGGKQALETVQ
RLLPVLCQAHGLTPAQVVAIASHDGGKQALETVQRLLPVLCQAHGLTPAQVVAIASNGGGRPAL
ESIVAQLSRPDPALAALTNDHLVALACLGGRPALDAVKKGLG*GS*GSYALGPYQISAPQLPAYNG
QTVGTFYYVNDAGGLESKVFSSGGPTPYPNYANAGHVEGQSALFMRDNGISEGLVFHNNPEGTC
GFCVNMTETLLPENAKMTVVPPEG*SGGS*TNLSDIIEKETGKQLVIQESILMLPEEVEEVIGNKP
ESDILVHTAYDESTDENVMLLTSDAPEYKPWALVIQDSNGENKIKML

Zebrafish G3890A Left TALE

LTPEQVVAIASNNGGKQALETVQRLLPVLCQAHGLTPEQVVAIASHDGGKQALETVQRLLPVLC
QAHGLTPAQVVAIASNIGGKQALETVQRLLPVLCQAHGLTPDQVVAIASNNGGKQALETVQRLL
PVLCQAHGLTPEQVVAIASNGGGKQALETVQRLLPVLCQAHGLTPEQVVAIASNIGGKQALETV
QRLLPVLCQAHGLTPDQVVAIASNNGGKQALETVQRLLPVLCQAHGLTPEQVVAIASHDGGKQA
LETVQRLLPVLCQAHGLTPAQVVAIASHDGGKQALETVQRLLPVLCQAHGLTPAQVVAIASNGG
GKQALETVQRLLPVLCQAHGLTPEQVVAIASNGGGKQALETVQRLLPVLCQAHGLTPEQVVAIA
SHDGGKQALETVQRLLPVLCQAHGLTPAQVVAIASHDGGKQALETVQRLLPVLCQAHGLTPAQV
VAIASNGGGKQALETVQRLLPVLCQAHGLTPEQVVAIASNGGGRPALE

Zebrafish G3890A Right TALE

LTPEQVVAIASNIGGKQALETVQRLLPVLCQAHGLTPDQVVAIASNGGGKQALETVQRLLPVLC
QAHGLTPEQVVAIASNIGGKQALETVQRLLPVLCQAHGLTPDQVVAIASNGGGKQALETVQRLL
PVLCQAHGLTPEQVVAIASNIGGKQALETVQRLLPVLCQAHGLTPDQVVAIASNNGGKQALETV
QRLLPVLCQAHGLTPEQVVAIASHDGGKQALETVQRLLPVLCQAHGLTPAQVVAIASHDGGKQA
LETVQRLLPVLCQAHGLTPAQVVAIASNGGGKQALETVQRLLPVLCQAHGLTPEQVVAIASNIG
GKQALETVQRLLPVLCQAHGLTPDQVVAIASNIGGKQALETVQRLLPVLCQAHGLTPDQVVAIA

SHDGGKQALETVQRLLPVLCQAHGLTPAQVVAIASNIGGKQALETVQRLLPVLCQAHGLTPDQV
VAIASHDGGKQALETVQRLLPVLCQAHGLTPAQVVAIASNGGGKQALETVQRLLPVLCQAHGLT
PEQVVAIASNGGGRPALE

Zebrafish G8892A Left TALE

LTPEQVVAIASNNGGKQALETVQRLLPVLCQAHGLTPEQVVAIASHDGGKQALETVQRLLPVLC
QAHGLTPAQVVAIASNGGGKQALETVQRLLPVLCQAHGLTPEQVVAIASNGGGKQALETVQRLL
PVLCQAHGLTPEQVVAIASNIGGKQALETVQRLLPVLCQAHGLTPDQVVAIASHDGGKQALETV
QRLLPVLCQAHGLTPAQVVAIASHDGGKQALETVQRLLPVLCQAHGLTPAQVVAIASNNGGKQA
LETVQRLLPVLCQAHGLTPEQVVAIASNIGGKQALETVQRLLPVLCQAHGLTPDQVVAIASHDG
GKQALETVQRLLPVLCQAHGLTPAQVVAIASHDGGKQALETVQRLLPVLCQAHGLTPAQVVAIA
SNIGGKQALETVQRLLPVLCQAHGLTPDQVVAIASHDGGKQALETVQRLLPVLCQAHGLTPAQV
VAIASHDGGKQALETVQRLLPVLCQAHGLTPAQVVAIASHDGGKQALETVQRLLPVLCQAHGLT
PAQVVAIASNGGGRPALE

Zebrafish G8892A Right TALE

LTPEQVVAIASHDGGKQALETVQRLLPVLCQAHGLTPAQVVAIASNIGGKQALETVQRLLPVLC
QAHGLTPDQVVAIASNNGGKQALETVQRLLPVLCQAHGLTPEQVVAIASNNGGKQALETVQRLL
PVLCQAHGLTPEQVVAIASNNGGKQALETVQRLLPVLCQAHGLTPEQVVAIASNGGGKQALETV
QRLLPVLCQAHGLTPEQVVAIASNGGGKQALETVQRLLPVLCQAHGLTPEQVVAIASNGGGKQA
LETVQRLLPVLCQAHGLTPEQVVAIASNGGGKQALETVQRLLPVLCQAHGLTPEQVVAIASNNG
GKQALETVQRLLPVLCQAHGLTPEQVVAIASNNGGKQALETVQRLLPVLCQAHGLTPEQVVAIA
SNIGGKQALETVQRLLPVLCQAHGLTPDQVVAIASNGGGKQALETVQRLLPVLCQAHGLTPEQV
VAIASNGGGKQALETVQRLLPVLCQAHGLTPEQVVAIASNIGGKQALETVQRLLPVLCQAHGLT
PDQVVAIASNIGGKQALETVQRLLPVLCQAHGLTPDQVVAIASNNGGKQALETVQRLLPVLCQA
HGLTPEQVVAIASHDGGKQALETVQRLLPVLCQAHGLTPAQVVAIASNGGGRPALE

Zebrafish G12833A Left TALE

LTPEQVVAIASNNGGKQALETVQRLLPVLCQAHGLTPEQVVAIASNNGGKQALETVQRLLPVLC
QAHGLTPEQVVAIASHDGGKQALETVQRLLPVLCQAHGLTPAQVVAIASNGGGKQALETVQRLL
PVLCQAHGLTPEQVVAIASNGGGKQALETVQRLLPVLCQAHGLTPEQVVAIASHDGGKQALETV
QRLLPVLCQAHGLTPAQVVAIASHDGGKQALETVQRLLPVLCQAHGLTPAQVVAIASNGGGKQA
LETVQRLLPVLCQAHGLTPEQVVAIASNGGGKQALETVQRLLPVLCQAHGLTPEQVVAIASNIG
GKQALETVQRLLPVLCQAHGLTPDQVVAIASHDGGKQALETVQRLLPVLCQAHGLTPAQVVAIA
SHDGGKQALETVQRLLPVLCQAHGLTPAQVVAIASNNGGKQALETVQRLLPVLCQAHGLTPEQV
VAIASHDGGKQALETVQRLLPVLCQAHGLTPAQVVAIASNGGGKQALETVQRLLPVLCQAHGLT
PEQVVAIASNGGGKQALETVQRLLPVLCQAHGLTPEQVVAIASHDGGKQALETVQRLLPVLCQA
HGLTPAQVVAIASNGGGRPALE

Zebrafish G12833A Right TALE

LTPEQVVAIASHDGGKQALETVQRLLPVLCQAHGLTPAQVVAIASHDGGKQALETVQRLLPVLC
QAHGLTPAQVVAIASNGGGKQALETVQRLLPVLCQAHGLTPEQVVAIASNIGGKQALETVQRLL
PVLCQAHGLTPDQVVAIASNIGGKQALETVQRLLPVLCQAHGLTPDQVVAIASNNGGKQALETV
QRLLPVLCQAHGLTPEQVVAIASNIGGKQALETVQRLLPVLCQAHGLTPDQVVAIASHDGGKQA
LETVQRLLPVLCQAHGLTPAQVVAIASHDGGKQALETVQRLLPVLCQAHGLTPAQVVAIASNIG
GKQALETVQRLLPVLCQAHGLTPDQVVAIASNIGGKQALETVQRLLPVLCQAHGLTPDQVVAIA
SNGGGKQALETVQRLLPVLCQAHGLTPEQVVAIASNNGGKQALETVQRLLPVLCQAHGLTPEQV
VAIASNNGGKQALETVQRLLPVLCQAHGLTPEQVVAIASNIGGKQALETVQRLLPVLCQAHGLT
PDQVVAIASNGGGRPALE

Zebrafish G14076A Left TALE

LTPEQVVAIASHDGGKQALETVQRLLPVLCQAHGLTPAQVVAIASHDGGKQALETVQRLLPVLC
QAHGLTPAQVVAIASNGGGKQALETVQRLLPVLCQAHGLTPEQVVAIASNGGGKQALETVQRLL
PVLCQAHGLTPEQVVAIASNNGGKQALETVQRLLPVLCQAHGLTPEQVVAIASHDGGKQALETV
QRLLPVLCQAHGLTPAQVVAIASNIGGKQALETVQRLLPVLCQAHGLTPDQVVAIASNNGGKQA
LETVQRLLPVLCQAHGLTPEQVVAIASNNGGKQALETVQRLLPVLCQAHGLTPEQVVAIASNIG
GKQALETVQRLLPVLCQAHGLTPDQVVAIASNGGGKQALETVQRLLPVLCQAHGLTPEQVVAIA
SNGGGKQALETVQRLLPVLCQAHGLTPEQVVAIASNGGGKQALETVQRLLPVLCQAHGLTPEQV
VAIASNGGGKQALETVQRLLPVLCQAHGLTPEQVVAIASNGGGKQALETVQRLLPVLCQAHGLT
PEQVVAIASHDGGKQALETVQRLLPVLCQAHGLTPAQVVAIASNGGGRPALE

Zebrafish G14076A Right TALE

LTPEQVVAIASNNGGKQALETVQRLLPVLCQAHGLTPEQVVAIASNNGGKQALETVQRLLPVLC
QAHGLTPEQVVAIASNGGGKQALETVQRLLPVLCQAHGLTPEQVVAIASNGGGKQALETVQRLL
PVLCQAHGLTPEQVVAIASNIGGKQALETVQRLLPVLCQAHGLTPDQVVAIASNGGGKQALETV
QRLLPVLCQAHGLTPEQVVAIASNGGGKQALETVQRLLPVLCQAHGLTPEQVVAIASNNGGKQA
LETVQRLLPVLCQAHGLTPEQVVAIASHDGGKQALETVQRLLPVLCQAHGLTPAQVVAIASNGG
GKQALETVQRLLPVLCQAHGLTPEQVVAIASNGGGKQALETVQRLLPVLCQAHGLTPEQVVAIA
SHDGGKQALETVQRLLPVLCQAHGLTPAQVVAIASNGGGKQALETVQRLLPVLCQAHGLTPEQV
VAIASNIGGKQALETVQRLLPVLCQAHGLTPDQVVAIASNNGGRPALE
